# Deep Learning-Enhanced Raman Microspectroscopy Enables Rapid Microbial Classification and Captures Phylogenetic Relationships

**DOI:** 10.3390/microorganisms14061311

**Published:** 2026-06-11

**Authors:** Beimin Liu, Zhenzhou Gu, Xianyang Xu, Weilai Lu, Tao Liu, Xueyan Gao, Xiaojing Chen, Yu Vincent Fu

**Affiliations:** 1 Medical Science and Technology Innovation Center, Shandong First Medical University & Shandong Academy of Medical Sciences, Jinan 250117, China; liubeimin01@163.com (B.L.); gaoxueyan@sdfmu.edu.cn (X.G.); 2 College of Life Sciences, South-Central Minzu University, Wuhan 430074, China; liutao@mail.scuec.edu.cn; 3 State Key Laboratory of Microbial Diversity and Innovative Utilization, Institute of Microbiology, Chinese Academy of Sciences, Beijing 100101, China; xuxianyang23@mails.ucas.ac.cn (X.X.); luweilai@163.com (W.L.); 4 College of Electrical and Electronic Engineering, Wenzhou University, Wenzhou 325035, China; 15094335799@163.com

**Keywords:** microbial classification, Raman spectroscopy, one-dimensional convolutional neural network, deep learning, phylogenetic tree

## Abstract

Microbial classification and taxonomic information are fundamental to microbiological studies. Raman microspectroscopy, a rapid and non-destructive single-cell analytical technique, captures intrinsic molecular fingerprints reflecting cellular biochemical composition, thereby enabling microbial classification at the single-cell level. However, current Raman-based classification frameworks allow accurate identification only for micro-organisms already represented in reference databases. These approaches often fail or yield errors for uncharacterized microorganisms. To address this limitation, we collected 6600 single-cell Raman spectra from 11 microbial species, including bacteria and fungi, and developed deep learning models for rapid classification. A hierarchical clustering (HC) framework based on Raman features extracted by a one-dimensional convolutional neural network (1D-CNN) was constructed and compared with phylogenetic trees derived from rRNA gene sequences. 1D-CNN achieved high classification performance with an overall accuracy of 99.7%. Notably, the Raman HC tree exhibited clear concordance with phylogenetic structures, particularly at the higher taxonomic levels. Validation using five independent unknown strains demonstrated that the Raman HC tree consistently positioned these strains near their closest phylogenetic relatives, in strong agreement with sequence-based analyses. Collectively, these findings highlight the potential of single-cell Raman spectroscopy with deep learning as an alternative and complementary framework for microbial taxonomic analysis, particularly for previously uncharacterized microorganisms.

## 1. Introduction

Accurate identification and classification of microorganisms constitute the foundation of most microbiological studies [1]. Early approaches to microbial taxonomy primarily relied on phenotypic characteristics, including morphological features, physiological and biochemical properties, and culture-based traits. For instance, colony morphology, cellular morphology, staining properties, and metabolic capabilities were commonly employed for microbial taxonomy [2]. With the advent of molecular biology, gene-based approaches and metagenomic sequencing technologies have become indispensable tools in microbial taxonomy [3]. For example, 16S rRNA gene sequencing is widely regarded as the gold standard for bacterial identification [4], while 28S rRNA genes and internal transcribed spacer (ITS) regions are commonly used for fungi, and comparisons with reference sequences in databases are used to achieve taxonomic identification and phylogenetic inference [5]. Whole genome sequencing (WGS) provides a more comprehensive framework for dissecting genetic diversity and evolutionary relationships among microorganisms at the strain level [6,7,8]. By comparing the genomic sequences (such as protein-coding gene sequences, non-coding regions, repetitive sequences, and structural variations) among different taxa, WGS enables a more precise elucidation of the phylogenetic relationships and evolutionary histories [5]. This approach not only supports strain-level identification but also facilitates the characterization of functional genes, antibiotic resistance genes, and virulence factors, thereby offering substantial value in microbial taxonomy and medical microbiology [9]. With the development of metagenomic sequencing technology, which eliminates the need for cultivation, researchers can uncover microbial identification at the strain level, as well as interactions between microorganisms and the environment.

Despite their accuracy, sequencing-based approaches face several limitations. Conserved gene (such as 16S rRNA gene) sequencing normally requires pure cultures of the microorganism, and the extraction, amplification, and sequencing of the target gene are labor-intensive and time-consuming. Moreover, it is difficult to apply these approaches at the single-cell level. In addition, the taxonomic resolution of 16S rRNA-based approaches is often inadequate for reliable strain-level identification. Although WGS provides higher resolution, it involves more complex sample preparation, longer processing times, and higher costs [9,10]. Importantly, it is estimated that 98% of microorganisms in nature cannot be cultured under laboratory conditions [11]. It is difficult to use the above methods for identification and taxonomic analysis when dealing with uncultured microorganisms. Although metagenomic sequencing circumvents the need for cultivation, it remains time-consuming, costly, and destructive, it is unsuitable for time-sensitive scenarios. Furthermore, current reference genome databases are heavily biased toward easily cultured and well-studied strains. Many uncultured microbes lack reference genomes, so they often cannot be reliably taxonomically classified [12,13].

Raman spectroscopy has emerged as a powerful analytical technique capable of probing the chemical composition of complex biological samples, including single cells [14,15,16,17]. Variations in cellular components, such as proteins, nucleic acids, lipids, polysaccharides, and metabolites, among microorganisms give rise to intrinsically distinct Raman spectral signatures, providing species-specific “fingerprinting” [18,19,20]. Based on these spectral differences, we can achieve rapid, culture-independent, non-destructive, label-free, and accurate microbial identification. The integration of Raman spectroscopy with deep learning has been successfully applied to the identification and characterization of diverse microorganisms, demonstrating robust performance [21,22,23,24].

However, a fundamental and long-standing limitation remains unresolved: existing Raman–AI frameworks rely heavily on predefined spectral libraries and can only accurately classify microorganisms that are already represented in reference datasets. When encountering previously uncharacterized microorganisms that are not included in any reference database, these approaches either fail or lead to incorrect classification. This limitation represents a major barrier to the broader application of Raman-based microbial analysis.

In conventional microbiology, unknown microorganisms are typically placed within a taxonomic framework through phylogenetic analysis based on conserved genetic markers. Closely related microorganisms generally exhibit high genomic sequence similarity, whereas more distantly related taxa display greater divergence. Phylogenetic analysis based on conserved genetic markers, such as the 16S rRNA gene for bacteria and ITS or 28S rRNA markers for fungi, is the standard approach for determining taxonomic relationships of unknown microorganisms by analyzing their clustering pattern relative to reference strains within a phylogenetic tree. In this study, the 16S rRNA gene was used for the phylogenetic analysis of bacteria, and the 28S rRNA gene was used for the phylogenetic analysis of fungi. Due to the unique Raman spectral signatures captured by the single-cell Raman spectroscopy technique, which are essentially determined by the cellular biochemical composition such as proteins, nucleic acids, lipids, polysaccharides, and metabolites. At the cellular level, biochemical composition is ultimately determined by genomic information. Therefore, phylogenetically related microorganisms may exhibit similarities in their biochemical profiles, although such biochemical similarity may not strictly reflect direct evolutionary lineage. Based on this rationale, we hypothesize that similarity patterns derived from Raman spectral features may be used to infer taxonomic relationships and assist in the classification of unknown microorganisms. Accordingly, constructing clustering trees based on Raman spectral similarity may provide a complementary framework for microbial classification and assist in the preliminary taxonomic placement of unknown microorganisms.

To test this hypothesis, we systematically investigated whether Raman spectral similarity cluster is consistent with phylogenetic relationships among different microbial strains. Bacteria and fungi were selected as representative microbial groups. Single-cell Raman spectra were acquired for each strain, and deep learning approaches were employed for microbial classification and spectral feature extraction. Hierarchical clustering (HC) was applied to features extracted by deep learning models to construct Raman HC trees. The resulting Raman HC trees were compared with phylogenetic trees generated using the neighbor-joining (NJ) method in order to evaluate the capacity of Raman spectroscopy to reflect microbial taxonomic relationships. Furthermore, we evaluated the practical utility of this framework by applying it to five previously unknown microbial strains and assessing their placement within the Raman HC tree. This study provides a proof-of-concept for leveraging single-cell Raman spectroscopy as a complementary approach for primary microbial taxonomic analysis and offers a potential pathway toward reference-free classification of unknown microorganisms.

## 2. Materials and Methods

### 2.1. Strains and Cultivation

For the deep learning model and Raman HC tree construction, a total of 22 microbial strains from 11 species were selected, including 20 fungal strains and 2 bacterial strains (Table 1). Among the fungi, *Saccharomyces paradoxus*, *Saccharomyces mikatae*, *Saccharomyces kudriavzevii*, *Saccharomyces arboricolus*, and *Saccharomyces eubayanus* each comprised three strains; *Pichia pastoris* comprised two strains; and *Saccharomyces cerevisiae*, *Candida albicans* and *Geotrichum candidum* each comprised one strain. The bacteria included *Acinetobacter baumannii* and *Klebsiella pneumoniae*, where each comprised one strain. All strains were obtained from a standard strain collection preserved in our laboratory. In addition, five unknown microbial strains were selected to validate the classification accuracy of the deep learning model by applying them to the Raman HC tree and assessing their placement.

Bacteria were cultured on LB agar plates at 37 °C for 12 h. The fungi, including *S. cerevisiae*, *S. paradoxus*, *S. mikatae*, *S. kudriavzevii*, *S. arboricolus*, *S. eubayanus*, were cultured on YPD agar plates at 25 °C for 48 h. The other fungi, *P. pastoris*, *G. candidum*, and *C. albicans* were cultured on YPD agar plates at 30 °C for 48 h. Cells were harvested after visible colonies formed and were subsequently used for Raman spectral acquisition and gene sequencing experiments. For the five unknown microbial strains, the four fungal strains were cultured using YPD, a universal fungal medium, and the one bacterial strain was cultured using LB, a universal bacterial medium.

### 2.2. Spectral Acquisition and Data Processing

After cultivation, microbial cells were washed three times with sterile water and resuspended in 1 mL of 0.9% NaCl solution. A 10 μL aliquot of the suspension was deposited onto a clean quartz substrate slide. The Raman spectrum of a single cell was acquired using a laser tweezers Raman spectroscopy system as previously described [19,21]. Specifically, after starting the system, the spectral resolution of the instrument was set to 1.5 cm^−1^, the laser power was 50 mW, and a 785 nm wavelength laser was used for spectral acquisition. Each acquisition was set with an integration time of 20 s, and a single cell was acquired once. We collected Raman spectra for 22 microbial strains. For each strain, 300 individual cells were selected, and a Raman spectrum was collected from each cell. Each single-cell Raman spectrum was treated as an independent analytical unit for downstream model training and evaluation. As a result, a total of 6600 single-cell Raman spectra were obtained for subsequent spectral analysis. For the external test dataset, all 27 microbial strains were cultured in independent batches separate from those used for model training and evaluation. For each strain, 50 single-cell Raman spectra were independently acquired and assigned exclusively to the external test set, ensuring that no spectra from the same experimental batch were shared between the training and external testing datasets.

The Raman spectra were converted to an ASCII file using Winspecs software (Version 2.6A). The “Ramanpro0.4.2” software package, developed in the R language, was employed for processing the Raman spectra. The preprocessing workflow included cosmic ray removal, background subtraction, smoothing, baseline correction, and normalization. For background subtraction, a blank spectrum collected from the quartz substrate containing 0.9% NaCl solution without microbial cells was used as the reference background spectrum. This procedure was performed to minimize spectral contributions from the quartz substrate and saline solution. After subtraction, no substantial substrate-related interference was observed within the Raman spectral region used for downstream analysis. Subsequently, spectral smoothing was performed using a Savitzky-Golay filter (version 0.4.2) with a window size of 11 points and a polynomial order of 3, followed by baseline correction using the Modpolyfit method to reduce fluorescence background and baseline drift. Finally, all spectra were normalized using the min–max normalization method prior to downstream analysis.

### 2.3. Gene Sequencing and Construction of Phylogenetic Tree

For microbial identification, bacterial strains were identified by 16S rRNA gene sequencing using primers 27F and 1492R, while fungal strains were identified by 28S rRNA (D1/D2 region) gene sequencing using primers NL1 and NL4. The primer sequences are listed in Table 2.

DNA was extracted from the microbial samples, and the bacterial 16S rRNA gene and fungal 28S rRNA gene were amplified by polymerase chain reaction (PCR). PCR products were stored at 4 °C for short-term use, verified by 1% agarose gel electrophoresis, and subsequently sent to BGI Genomics (Beijing, China) for sequencing.

For taxonomic identification of the five unknown microbial strains, sequence similarity analysis was performed using the BLASTn algorithm against the Core nucleotide database within the rRNA/ITS databases (NCBI). Bacterial strains were identified based on 16S rRNA gene sequences, whereas fungal strains were identified based on 28S rRNA gene sequences. Taxonomic assignment was determined according to the top-scoring reference sequence, considering sequence identity and query coverage.

The phylogenetic tree was constructed using MEGA 11 software [25]. Multiple sequence alignment (MSA) was performed using the ClustalW method to globally align all sequences [26], and the NJ method was selected to construct the phylogenetic tree [27]. Evolutionary distances were calculated using the nucleotide p-distance substitution model, including both transitions and transversions. Bootstrap analysis with 1000 replicates was performed to assess support for each branch.

### 2.4. Distance Calculation and Hierarchical Clustering

Spectral data were processed using R (version 4.3.2). First, spectral data were read from each CSV file, and the numerical values were extracted as feature vectors. All samples were then combined into a matrix, and pairwise differences between samples were calculated using distance metrics such as Euclidean distance or Manhattan distance [28]. HC using the complete-linkage method [29] was applied to construct a clustering tree, with results typically represented as a dendrogram [30]. After performing 1000 repetitions and HC iterations, the optimal clustering tree was output.

### 2.5. Machine Learning Techniques

In Raman spectral data, meaningful information is intermingled with irrelevant noise, making it difficult to reveal the true differences between different samples through direct observation of the spectral or spectrogram data. To address this issue, we introduced multiple classifiers to perform feature extraction and comprehensive analysis on the standardized Raman spectral data.

Support vector machine (SVM) [31] with a radial basis function (RBF) kernel achieves the accurate classification of complex nonlinear boundaries by nonlinearly mapping the data into a high-dimensional space and finding the optimal hyperplane within it.

Least absolute shrinkage and selection operator (LASSO) applies L1 regularization [32] to automatically perform variable selection during regression modeling, shrinking the coefficients of irrelevant features to zero, thereby enhancing model sparsity and interpretability [33].

Multilayer perceptron (MLP) is a feedforward neural network that, through multiple layers of nonlinear transformations and the backpropagation algorithm, can approximate complex nonlinear functions with arbitrary precision [34].

The one-dimensional convolutional neural network (1D-CNN) [35] used in this study consists of two convolutional layers, two pooling layers, and a fully connected layer. For model development, the Raman spectral dataset was randomly divided into training (70%) and test (30%) sets using a stratified splitting strategy to preserve the proportional distribution of each microbial strain across the two datasets. The held-out test set was not involved in model training and was used exclusively for model evaluation. Standardized Raman spectral data are first input into the model. The first convolutional layer uses 32 kernels of size 3 to extract basic peak features, followed by a max-pooling layer for downsampling to reduce dimensionality and enhance feature stability. The second convolutional layer increases the number of kernels to 64, integrating information across different spectral bands, and is followed by a second pooling operation to further compress the feature space. The resulting high-dimensional features are then mapped to a 128-dimensional latent feature space via a fully connected layer, with a dropout rate of 0.5 applied to reduce overfitting. Finally, classification outputs are normalized using a Softmax function. During training, the cross-entropy loss function is employed as the optimization objective, and the Adam optimizer is used for parameter updates with a learning rate of 0.001. Weight decay (0.001) is introduced to improve model generalization [36]. The network is trained for 50 epochs, and a fixed random seed is used to ensure reproducibility.

In addition, SHapley Additive exPlanations (SHAP) values were calculated for the 1D-CNN model to approximate the contribution of each wavelength feature to the model output based on gradient information [37]. SHAP is derived from the Shapley value in game theory, and its core idea is to decompose the model prediction into the contributions of each input feature [38]. In this study, the Gradient Explainer was used to interpret the 1D-CNN model. SHAP values were calculated for each wavenumber point across different samples, and the mean absolute value was further computed to evaluate the importance of each spectral region in the overall classification task [37]. Larger SHAP values indicate a stronger influence of that wavenumber on the model output. This metric quantifies the average contribution of each wavenumber to the classification task. By ranking this metric, the top 20 most contributing wavenumber regions were selected for subsequent analysis and visualization.

## 3. Results

### 3.1. Single-Cell Morphology and Raman Spectra Acquisition

**Figure 1:**
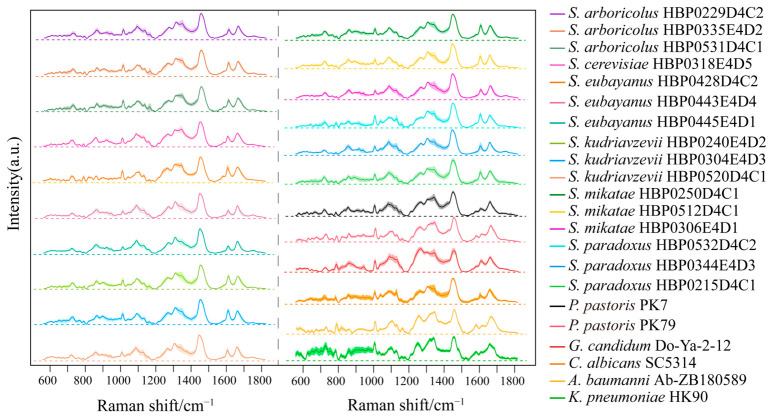
Average Raman spectra of 22 microbial strains. The averaged Raman spectra of the 22 strains were located within the 550–1850 cm^−1^ wavenumber range. The mean Raman spectra are shown by the solid line, and the standard deviations are represented by the shadow, and the dotted line represents the baseline of the average spectrum.

Single microbial cells were captured by laser tweezers and their Raman spectra were acquired using the laser tweezers Raman spectroscopy system. The typical cell morphologies of 11 different species observed under microscope are shown in Figure S1. In contrast to the distinct morphological differences typically observed among plant and animal species, microbial cells often exhibit highly similar cellular morphologies, especially within fungi or within bacteria, making accurate microbial identification based solely on visual features impossible. The average Raman spectra of the 22 strains are shown in Figure 1.

### 3.2. Construction of Raman HC Tree

First, we performed distance calculations on the standardized Raman spectra and constructed a Raman HC tree to explore whether the resulting classification pattern showed any concordance with the phylogenetic relationships. We represented the standardized Raman spectrum of each strain as a high-dimensional vector, in which each dimension corresponded to the spectral intensity at a specific wavenumber. By calculating the differences in peak intensities between strains, a distance matrix was constructed, which was subsequently used to generate a Raman HC tree.

**Figure 2:**
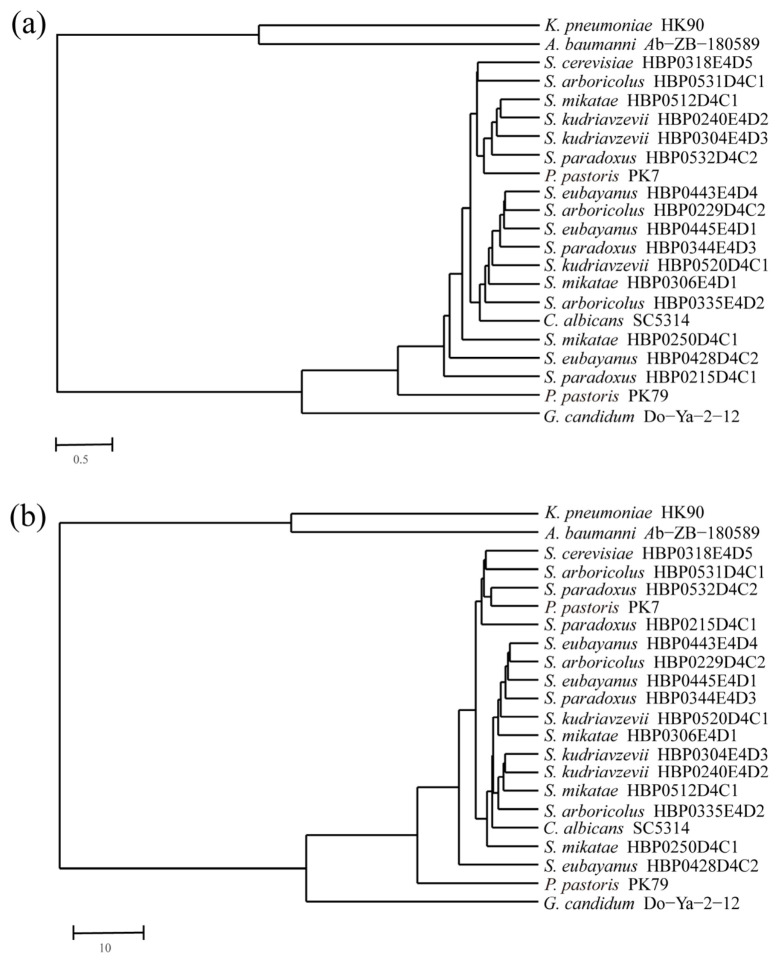
Raman HC tree based on the standardized Raman spectra. (**a**) Raman HC tree constructed using Euclidean distance; (**b**) Raman HC tree constructed using Manhattan distance.

As shown in Figure 2, the Raman HC trees were constructed based on the Euclidean and Manhattan distances, respectively. Unfortunately, the Raman HC tree directly derived from standardized spectra showed limited taxonomic resolution, in which even microbial strains from the same species failed to cluster into adjacent branches. The results implied that high-dimensional Raman spectral data may be affected by substantial background noise and information redundancy, rendering metrics simply based on the standardized spectra inadequate for capturing the intrinsic similarities and differences among microbial strains.

### 3.3. Clustering Microbial Strains by Extracted Raman Spectral Features Using SVM, LASSO and MLP

Since the direct use of standardized Raman spectra to construct a Raman HC tree yielded poor classification results that were not consistent with the phylogenetic relationships at all, we used machine learning approaches to extract effective features from the standardized spectra before performing microbial clustering to minimize the effects of background noise and redundancy. If the machine learning model can classify the microbial strains with high accuracy, it is likely that the machine learning approach extracted effective features of the Raman spectrum.

**Figure 3:**
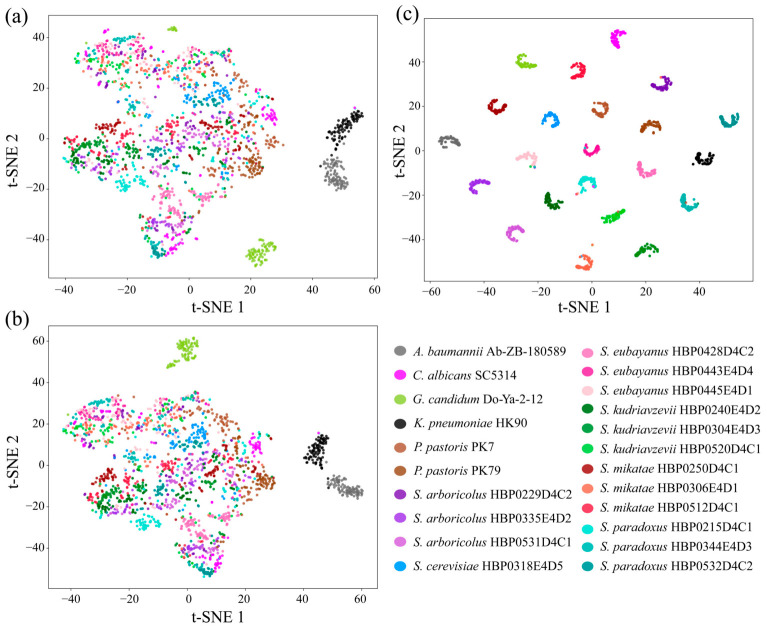
Visualization of clustering under different Raman feature extraction methods. (**a**) t-SNE visualization of standardized Raman spectra after SVM feature extraction. (**b**) t-SNE visualization after LASSO feature selection followed by SVM classification. (**c**) t-SNE visualization of features extracted by MLP from raw Raman spectral data. Different colors represent different microbial strains.

We first used SVM, a typical machine learning approach, to classify microbial strains. The spectral dataset was split into 70% training and 30% testing data to construct an SVM model. The overall classification accuracy of SVM was 95.0%, and the classification results are shown in the confusion matrices in Figure S2. A t-distributed stochastic neighbor embedding (t-SNE) was then applied for nonlinear dimensionality reduction and visualization of the clustering results. As shown in Figure 3a, clear overlap existed among several strain clusters, with cluster boundaries appearing blurred. The unclear cluster boundaries might have been caused by the sole dependence on shallow statistical features, indicating that SVM did not capture effective features from the Raman spectra.

To further enrich the effective features of the Raman spectral data, we introduced LASSO to perform preliminary screening of the spectral data based on L1 regularization. By incorporating L1 regularization into the loss function, LASSO imposes a sparsity constraint on high-dimensional spectral features, shrinking coefficients of less important features to zero, thereby achieving both feature selection and dimensionality reduction. The regularized features were then input into an SVM to construct a classification model, followed by t-SNE visualization. The overall classification accuracy of the LASSO-SVM model was 95.8% (Figure S3), a little bit higher than the SVM model. However, as shown in Figure 3b, there remained a certain degree of overlap among the strain clusters, indicating that LASSO still struggled to fully capture the complex feature structure in Raman spectra. This may be because LASSO is essentially a linear feature selection method, which makes it difficult to select features based on nonlinear relationships in high-dimensional spectral data. Therefore, although the introduction of LASSO reduced the data dimensionality to some extent and improved interpretability, it still could not accurately distinguish the spectral differences among different strains.

We also replaced the RBF-kernel SVM with an MLP classifier. As a feedforward neural network architecture, MLP can gradually extract high-dimensional features from spectral data through multilayer nonlinear transformations. The overall classification accuracy of the MLP model increased to 99.1% (Figure S4). The t-SNE visualization of the cluster results showed that most strains formed relatively independent clusters, with more compact within-cluster distributions and significantly increased between-cluster distances. However, some cluster overlap persisted among closely related strains (Figure 3c).

### 3.4. Clustering Strains by Raman Spectral Feature Extraction Using 1D-CNN

To improve the clustering capability on microbial strains, a 1D-CNN was introduced for deep feature extraction and classification analysis. Compared with traditional fully connected networks, the 1D-CNN can automatically extract local features by sliding convolutional kernels along the spectral dimension, better capturing correlations between adjacent wavenumbers in the Raman spectra.

The confusion matrix of the 1D-CNN model showed that most of the 22 microbial strains were correctly classified (Figure S5A), and the classification accuracy increased to 99.7%. During model training, the loss function decreased rapidly and gradually stabilized over successive epochs (Figure S5B), with no significant oscillations observed, indicating that the model architecture and parameter settings are reasonably designed, and no overfitting occurred.

**Figure 4:**
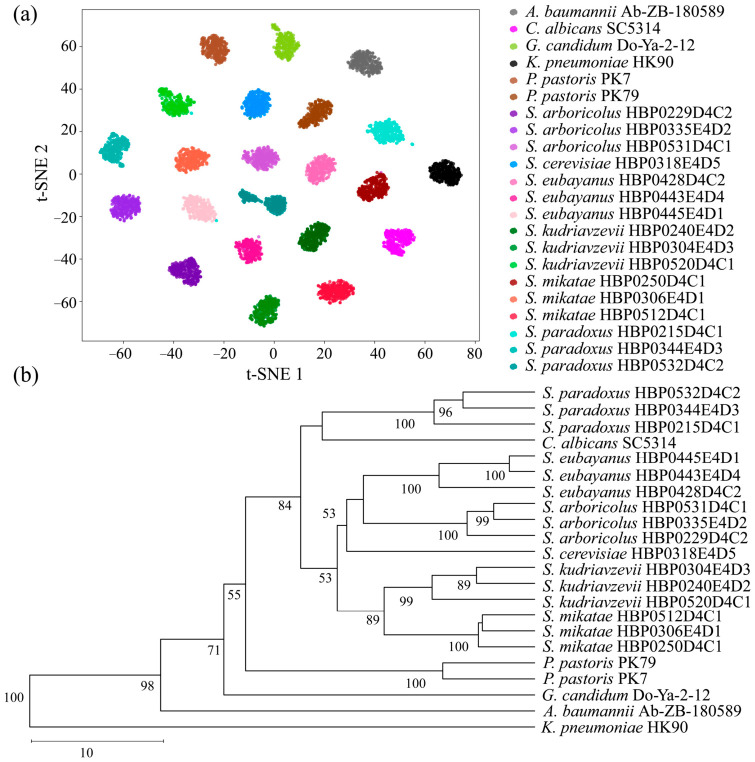
Classification of 22 strains by the 1D-CNN model and Raman HC tree based on the features extracted by 1D-CNN. (**a**) t-SNE visualization of 22 strains using features extracted by the 1D-CNN model. (**b**) Raman HC tree of 22 strains constructed based on features extracted by the 1D-CNN model.

By further combining the t-SNE dimensionality reduction results, the high-level features extracted by the 1D-CNN form clearly separated clusters in the low-dimensional space (Figure 4a). Clusters from each strain showed high intra-cluster compactness and pronounced inter-cluster separation, indicating that the 1D-CNN model extracts the Raman spectral features of each strain to successfully discriminate them.

Next, we further constructed a Raman HC tree based on these extracted features by the 1D-CNN model. The feature vectors representing each strain in the high-dimensional feature space were extracted from the trained 1D-CNN model. The Euclidean distances between different strains were calculated, and a complete-linkage HC method was employed to construct the Raman HC tree. The resulting Raman HC tree of the 22 strains is shown in Figure 4b, where the bacteria and fungi were divided into two distinct major branches. Within the major fungal branch, the fungal strains from the same species formed closed distance hierarchical branches in the dendrogram, and distinct separation trends were observed between different species.

### 3.5. Comparison of the Positions of Unknown Strains in the Raman HC Tree and the Phylogenetic Tree

To evaluate whether unknown microorganisms could be accurately classified and appropriately positioned within the Raman HC tree, we analyzed five previously uncharacterized microbial strains using the 1D-CNN model and compared their placement with phylogenetic relationships inferred from DNA sequencing.

**Figure 5:**
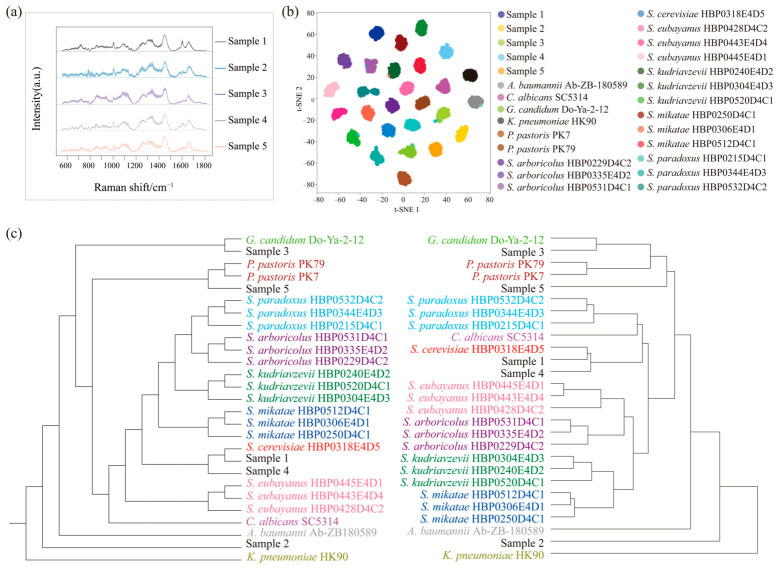
The classification of five unknown microbial strains (**a**) The average single-cell Raman spectrum of the five microbial samples; (**b**) t-SNE visualization of five unknown strains clustered by the 1D-CNN classification model. (**c**) Comparation between the phylogenetic tree and Raman HC tree. The left panel shows an NJ tree constructed from DNA sequencing data, while the right panel shows a Raman HC tree generated using Raman spectral feature distances extracted by the 1D-CNN model.

Morphological characterization of these five unknown strains was first performed (Figure S6). Among them, Sample 2 (Figure S6B) exhibited a markedly smaller cell size compared to the other samples, whereas Sample 3 (Figure S6C) clearly displayed a rod-shaped morphology. Single-cell Raman spectra were subsequently acquired for all five strains. As shown in Figure 5a, the average spectra exhibited only subtle variations, with overall spectral differences remaining relatively minor.

The five unknown strains were then classified using the 1D-CNN model, achieving an average accuracy of 99.5%, as illustrated by the confusion matrix (Figure S7). t-SNE visualization further demonstrated that all five samples formed well-separated clusters, without overlap with other strains in the dataset (Figure 5b), indicating strong discriminative performance of the model.

To further assess their taxonomic positioning, pairwise distances derived from the 1D-CNN features were used to construct a Raman HC tree incorporating both the five unknown strains and the previously analyzed 22 reference strains. In parallel, all 27 strains were subjected to DNA sequencing to generate an NJ tree.

A direct comparison between the Raman HC tree and the NJ tree is presented in Figure 5c. Overall, the Raman HC tree exhibited a high degree of concordance with the phylogenetic tree, despite minor differences in branching topology. Notably, all five unknown strains were positioned in close proximity to their respective phylogenetic relatives in the Raman HC tree, consistent with sequence-based identification, resulting in 100% correct species-level classification.

As shown in Table 3, based on BLAST (NCBI) sequence analysis (https://blast.ncbi.nlm.nih.gov, accessed on 4 June 2026) against the Core nucleotide database within the rRNA/ITS databases, Samples 1 and 4 were identified as *S. cerevisiae*, and were clustered together in the Raman HC tree with *S. cerevisiae* HBP0318E4D5; Sample 2 was identified as *Escherichia coli*, and was correctly positioned in the bacterial branch in the Raman HC tree; Sample 3 was identified as *G. candidum*, and was grouped together with *G. candidum* Do-Ya-2-12 in the Raman HC tree; Sample 5 was identified as *Cryptococcus neoformans*, and was separately classified into a branch in the Raman HC tree, adjacent to *P. pastoris*. The clustering results of these five samples in the Raman HC tree are consistent with the results of the NJ tree. All of these results prove the accuracy of this classification framework based on Raman spectroscopy.

**Figure 6:**
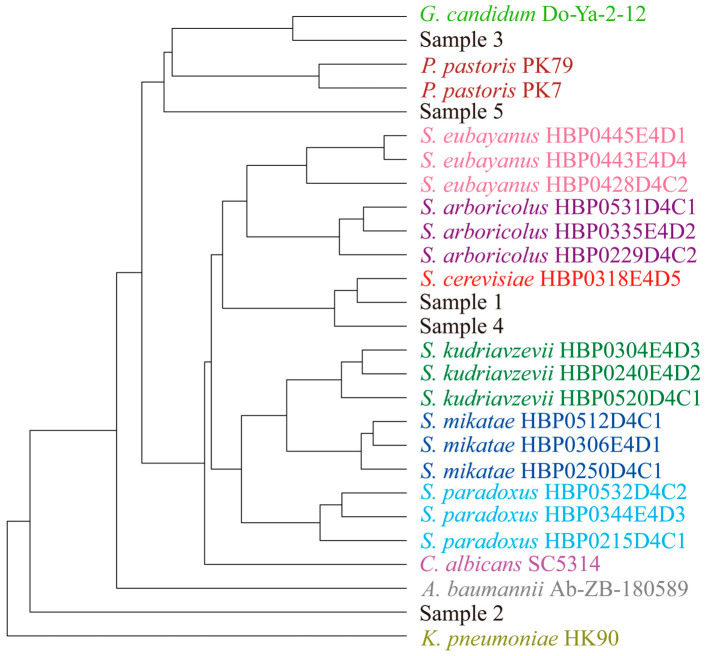
The HC tree constructed using the independent test set. For each of the 27 microbial strains, 50 single-cell Raman spectra were acquired from independently cultured batches. Different colors in the tree represent different species. The HC tree showed clear microbial clustering patterns and substantial consistency with sequence-based relationships.

Furthermore, we evaluated the robustness and generalizability of the 1D-CNN model using an independent external test dataset. All 27 microbial strains were re-cultured in independent batches separate from those used for model training. For each strain, 50 single-cell Raman spectra were newly and independently acquired and assigned exclusively to the external test set. In this independent validation, the model achieved an overall classification accuracy of 94.3%, as shown in the confusion matrix in Figure S8. In addition, the hierarchical clustering (HC) tree continued to show clear taxonomic clustering patterns and substantial consistency with sequence-based relationships (Figure 6).

Taken together, these results demonstrate that similarity patterns derived from Raman spectral features can reliably reflect underlying taxonomic relationships and provide a robust framework for the classification of previously unknown microorganisms.

### 3.6. Using SHAP to Explain the Important Features Extracted by 1D-CNN

**Figure 7:**
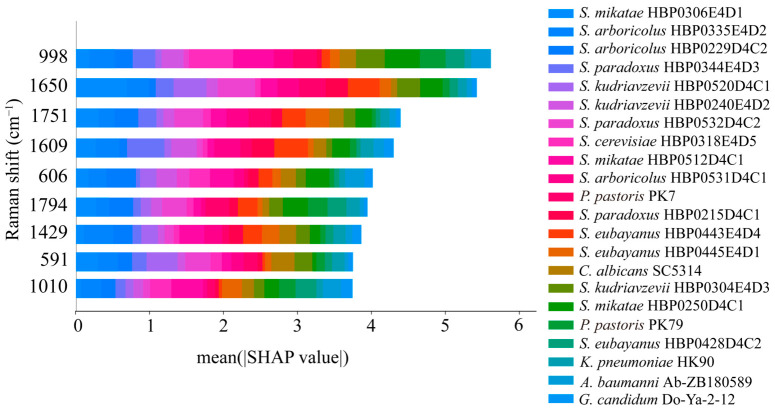
Important features distributing to 1D-CNN model performance based on SHAP analysis. The x-axis represents the mean absolute SHAP value, indicating the average contribution of each wavenumber to the 1D-CNN model performance; different colors correspond to different microbial strains.

In order to reveal the important spectral features extracted by the 1D-CNN model for classification, we applied SHAP to quantify the contribution of individual wavenumber variables to the model output. In Figure 7, the variation along the x-axis, representing SHAP values, reflects the contribution of each Raman wavenumber to the classification performance of the 1D-CNN model across the 22 strains. Table 4 summarizes the assignments of characteristic Raman peaks based on SHAP analysis of the 1D-CNN model. Notably, the SHAP value at 998 cm^−1^ was the highest, indicating that this spectral peak played a critical role in distinguishing between different microbial strains. This peak was primarily associated with C–C stretching in β-sheet structures of proteins and =CH bending in lipids, suggesting that these biochemical components might serve as important discriminative features.

## 4. Discussion

This study aimed to address a key limitation of current Raman–AI approaches in the classification of previously unknown microorganisms. Analogous to phylogenetic tree construction in conventional microbial taxonomy, we employed a 1D-CNN to extract high-level spectral features and subsequently constructed a Raman HC tree based on the Euclidean distances among these features. The resulting Raman HC tree exhibited substantial concordance with the phylogenetic trees constructed from conserved genetic markers. In validation experiments using five unknown microbial strains, their positions within the Raman HC tree were highly consistent with those observed in the NJ tree. Specifically, each strain was clustered in close proximity to its phylogenetically closest relative, as determined by sequence-based analysis. These findings demonstrate that Raman spectroscopy, when integrated with deep learning, provides a rapid and effective framework for single-cell-level microbial classification, with the capacity to infer taxonomic relationships even for previously uncharacterized organisms.

### 4.1. Performance Comparison of Different Machine Learning Methods

In this study, various methods including SVM, MLP, and 1D-CNN were employed to model the spectral data, and the accuracies of the classification results were compared.

First, as shown in the t-SNE plot (Figure 3a), even with the RBF kernel, SVM still struggled to effectively capture the nonlinear high-dimensional features in the Raman spectral data. Although LASSO preprocessing alleviated the impact of noise and redundant information to some extent, as a linear method, it cannot effectively handle the nonlinear relationships in spectral data, thus providing only very limited improvement in classification performance (Figure 3b). In contrast, the MLP model performed multilayer nonlinear transformations and achieved more effective feature extraction than the aforementioned methods; the t-SNE visualization (Figure 3c) showed that most strains were clearly distinguished. However, some strains were still misclustered, indicating that while MLP has a strong ability to discriminate spectral data with nonlinear features, it may still extract incorrect features for strains with very subtle spectral differences, leading to inaccurate classification results.

In contrast, the 1D-CNN model demonstrated strong performance in the Raman spectral classification tasks, not only significantly improving the classification accuracy but also achieving a clear separation of categories in the feature space, thereby providing a solid foundation for subsequent phylogenetic analyses using spectral features. From a methodological perspective, the superiority of 1D-CNN over SVM and MLP can be attributed to several key factors: First, the convolution operation, through its local receptive field mechanism, captures correlations between adjacent wavenumbers, enabling the identification of biologically meaningful peak combinations [35]. Second, the weight-sharing mechanism reduces the number of model parameters and improves generalization Finally, the multi-layer nonlinear mappings allow the model to extract critical features directly from the raw spectra, thereby significantly enhancing classification performance. This indicates that compared to linear models such as LASSO, using nonlinear models to process our spectral data achieved better results. Therefore, although methods like LDA and QDA, which are based on linear or quadratic discriminant assumptions, are commonly used benchmark methods in spectral classification, for our data, adopting nonlinear models for spectral discrimination is a more reasonable strategy.

The deep features extracted by the 1D-CNN model were further used to construct a Raman HC tree. The results showed that strains of the same species were successfully clustered into the same branches, suggesting that the microbial composition information obtained through Raman spectroscopy has a significant and repeatable consistency at the species level and in relation to the phylogenetic structure. Notably, high classification accuracy reflects the ability of the 1D-CNN model to discriminate microbial strains based on Raman spectral features, whereas phylogenetic inference concerns whether Raman-derived spectral similarities show concordance with sequence-based taxonomic relationships. Unlike conventional Raman–AI approaches, the present framework emphasizes the preservation of taxonomically relevant spectral relationships to support the classification of unknown microorganisms.

The SHAP analysis results indicate that except for the wavenumber at 998 cm^−1^, several high-contribution spectral regions, including 1751 cm^−1^ and 1794 cm^−1^, overlapped with the Raman bands previously reported for lipid-related carbonyl stretching and oxidized lipid species. These model-important spectral regions may reflect biologically relevant biochemical variation among microorganisms and could contribute to the discriminative performance of the 1D-CNN model. When combined with spectral peak assignments, these findings indicate that intensity variations in specific Raman regions may contribute to microbial discrimination by the model.

### 4.2. Raman Spectroscopy for Classifying Unknown Microorganisms

In this study, five unknown microbial strains were selected to evaluate the ability of the proposed framework to classify previously uncharacterized microorganisms. Neither morphological observation nor direct comparison of average Raman spectra was sufficient to determine their taxonomic identities. However, by extracting spectral features using the 1D-CNN model and constructing a Raman HC tree, the five unknown strains were positioned in a manner highly consistent with their placement in the NJ tree. For example, in the Raman HC tree, Sample 3 was clustered together with *G. candidum* Do-Ya-2-12, indicating that they are similar in the spectral features extracted by the deep learning model. This suggests that they share comparable cellular biochemical compositions, demonstrating that the classification framework can accurately cluster strains belonging to the same species. Sample 5 was located adjacent to *P. pastoris* in the Raman HC tree, indicating a certain degree of similarity between their Raman spectral features as extracted by the model. However, Sample 5 formed an independent branch rather than clustering within *P. pastoris*, suggesting that the model identified substantial differences in the cellular biochemical compositions reflected by their spectra. These differences were significant enough to prevent Sample 5 from being classified as *P. pastoris*. A consistent topology was also observed in the NJ tree, where Sample 5 occupied a similar position. According to the DNA sequencing results, Sample 5 was identified as *C. neoformans*, confirming that it does not belong to *P. pastoris*. This further demonstrates that the proposed classification framework can effectively distinguish between different species. The introduction of the independent test set supported the model’s excellent generalization ability and reliability. Overall, these results indicate that the Raman HC tree provides accurate classification for unknown microorganisms, and this classification result is consistent with the results obtained through sequencing identification. The molecular fingerprints derived from Raman spectra are capable of capturing biologically meaningful similarities, and to a certain extent, reflecting the taxonomic relationships among microorganisms.

### 4.3. Potential for Direct Single-Cell Taxonomic Inference Under the Microscope

Overall, our results demonstrate that Raman spectral data, when processed using the 1D-CNN model, enable the accurate classification of unknown microorganisms and provide a complementary approach to conventional microbial taxonomy. This framework offers the potential to infer taxonomic information directly at the single-cell level under the microscope in a rapid, non-destructive, and label-free manner, without requiring prior inclusion in reference databases.

First, as most microorganisms still require cultivation for taxonomic identification, Raman spectroscopy could serve as a preliminary classification tool prior to cultivation [48], even for completely unknown species, at the single-cell level. Second, due to its minimal invasiveness [49], Raman spectroscopy allows for initial screening without compromising cell viability, enabling the downstream cultivation of selected microorganisms while reducing redundant experimental workload. This is particularly advantageous for rare or valuable samples. In contrast to destructive methods such as metagenomic sequencing, Raman-based analysis preserves the possibility of subsequent pure culture. Finally, the rapid acquisition of Raman spectra provides a practical advantage in time-sensitive scenarios where conventional sequencing methods may be too slow.

### 4.4. The Limitations of This Study

Despite these promising findings, several limitations should be acknowledged. First, the dataset included a relatively limited number of strains and taxonomic groups, which may constrain the generalizability of the model. Although the loss curve indicates that the model does not overfit the current samples, the limited diversity of the training data may have led the model to become somewhat specialized to the current data distribution, which could still result in generalization errors when faced with more complex and diverse samples. Moreover, although five previously unknown microbial strains were used for additional evaluation, a large-scale independent external validation dataset was not included in the present study, which may limit the assessment of model robustness across different experimental settings and microbial diversity. Meanwhile, the microorganisms covered in this study were primarily from the Saccharomycetales, with only a limited number of bacterial taxa included, while more complex fungal groups—such as filamentous ascomycetes and basidiomycetes—were not involved. This limitation affects the generalizability of the model to some extent, particularly in the classification task of complex fungi. Therefore, datasets with broader taxonomic coverage are needed to further examine whether Raman spectral similarity can consistently reflect biological relationships in complex evolutionary lineages. Second, Raman spectra primarily reflect cellular biochemical composition, which can be influenced by environmental conditions, growth phase, and experimental variability [50]. Therefore, future research should incorporate Raman spectroscopy data obtained under different culture media, growth stages, and experimental conditions, in order to enhance the robustness of the model and increase its general applicability in practical applications. Third, although a degree of concordance between Raman-based clustering and phylogenetic relationships was observed, this does not imply that Raman spectroscopy can replace sequence-based phylogenetic inference.

Importantly, the current framework does not constitute a true open-set recognition system and is not intended for definitive microbial identification. Instead, it aims to provide preliminary taxonomic placement based on Raman spectral similarity. Future work should focus on expanding dataset diversity and developing more robust feature extraction strategies to mitigate environmental and experimental variability, thereby enhancing model robustness. In addition, integrating emerging approaches such as open-set recognition, out-of-distribution detection, and uncertainty estimation would allow the framework to better recognize microorganisms that are not represented in the existing reference dataset [51,52], rather than forcing them into predefined classes. These methodological improvements would further enhance the applicability of the proposed Raman spectroscopy–deep learning framework in real-world scenarios, where unknown, rare, or previously uncharacterized microorganisms are frequently encountered.

## 5. Conclusions

In conclusion, this study demonstrates that Raman spectroscopy combined with deep learning provides a rapid and effective framework for single-cell microbial classification. Deep features extracted by the 1D-CNN model captured biologically meaningful variations in cellular composition and enabled the construction of a Raman HC tree based on Raman spectral similarity. Although the Raman HC tree showed only partial concordance with sequence-based phylogeny, reflecting biochemical similarity rather than true evolutionary relationships, it nevertheless provided complementary phenotypic information for comparing microbial relationships. These findings suggest that Raman spectroscopy may serve as a promising non-invasive approach for microbial classification and preliminary taxonomic placement of unknown microorganisms relative to reference strains. With further methodological refinement and spectral dataset expansion, this approach may hold considerable potential for applications in clinical diagnostics, environmental monitoring, and high-throughput microbial screening.

## Figures and Tables

**Table 1:** Microbial sample strains and species.

Kingdom	Genus	Species	Strain
Fungi	*Saccharomyces*	*Saccharomyces paradoxus*	HBP0532D4C2
			HBP0344E4D3
			HBP0215D4C1
		*Saccharomyces mikatae*	HBP0250D4C1
			HBP0512D4C1
			HBP0306E4D1
		*Saccharomyces kudriavzevii*	HBP0240E4D2
			HBP0304E4D3
			HBP0520D4C1
		*Saccharomyces arboricolus*	HBP0229D4C2
			HBP0335E4D2
			HBP0531D4C1
		*Saccharomyces eubayanus*	HBP0428D4C2
			HBP0443E4D4
			HBP0445E4D1
		*Saccharomyces cerevisiae*	HBP0318E4D5
	*Pichia*	*Pichia pastoris*	PK7
			PK79
	*Candida*	*Candida albicans*	Do-Ya-2-12
	*Geotrichum*	*Geotrichum candidum*	SC5314
Bacteria	*Acinetobacter*	*Acinetobacter baumannii*	Ab-ZB180589
	*Klebsiella*	*Klebsiella pneumoniae*	HK90

**Table 2:** Primer sequences.

Primers	Target Gene	Sequence	bp
27F	16S rRNA	5′-AGAGTTTGATCCTGGCTCA-3′	~1500
1492R	16S rRNA	5′-GGTTACCTTGTTACGACTT-3′	~1500
NL1	28S rRNA (D1/D2 region)	5′-GCATATCAATAAGCGGAGGAAAAG-3′	~600
NL4	28S rRNA (D1/D2 region)	5′-GGTCCGTGTTTCAAGACGG-3′	~600

**Table 3:** Sequence-based identification of unknown microbial strains.

Sample ID	Scientific Name	Sequence Identity (%)	Query Coverage (%)
Sample 1	*Saccharomyces cerevisiae*	99.47	99.00
Sample 2	*Escherichia coli*	100.00	100.00
Sample 3	*Geotrichum candidum*	100.00	100.00
Sample 4	*Saccharomyces cerevisiae*	99.83	98.00
Sample 5	*Cryptococcus neoformans*	100.00	100.00

**Table 4:** Assignment of characteristic Raman peaks based on SHAP analysis of the 1D-CNN model features.

Raman Peak (cm^−1^)	Assigned Molecular Component	Reference
591 (589)	Symmetric stretching vibration of $v_{4}\;{PO}_{4}^{3-}$ (phosphate of HA) Glycerol	[39]
606	Glycerol	[40]
998	C-C stretching b-sheet (proteins) =CH bending (lipids)	[41]
1010 (1008)	Phenylalanine *v*(CO), *v*(CC), δ(OCH), ring (polysaccharides, pectin)	[42]
1429	CH_2_ scissoring vibration (lipid band)	[42,43]
1609	Cytosine (NH_2_)	[44]
1650	(C=C) Amide I Protein amide I absorption Amide I	[45]
1751	C=O (lipid in normal tissues) *v*(C=C) lipids, fatty acids	[46,47]
1794	Anhydride C=O stretching/oxidized lipids	[40]

## Data Availability

The original contributions presented in this study are included in the article. Further inquiries can be directed to the corresponding authors.

## Supplementary Materials

The following supporting information can be downloaded at: https://www.mdpi.com/article/10.3390/microorganisms14061311/s1, Figure S1: Morphological images of 11 different microbial species made at 1000× magnification; Figure S2: Test set confusion matrix of normalized Raman spectra of 22 strains based on the SVM classifier, the classification accuracy reached 95.0%; Figure S3: Test set confusion matrix of normalized Raman spectra of 22 strains based on L1 regularization preprocessing and SVM, the classification accuracy reached 95.8%; Figure S4: Test set confusion matrix of standardized Raman spectra of 22 strains based on MLP, the classification accuracy reached 99.1%; Figure S5: Test set confusion matrix and evaluation results of the 1D-CNN model; Figure S6: Morphological images of five unknown microbial strains captured at 1000× magnification; Figure S7: Classification confusion matrix results of the five unknown microbial strains on the 1D-CNN model, the classification accuracy reached 99.5%; Figure S8: The classification confusion matrix results of the 27 microbial strain independent spectral test set on the 1D-CNN model show that the classification accuracy reached 94.3%.

## Abbreviations

The following abbreviations are used in this manuscript:

HC	Hierarchical Clustering
NJ	Neighbor-Joining
SVM	Support Vector Machine
LASSO	Least Absolute Shrinkage and Selection Operator
MLP	Multilayer Perceptron
1D-CNN	One-Dimensional Convolutional Neural Network
SHAP	SHapley Additive exPlanations
RBF	Radial Basis Function
t-SNE	t-Distributed Stochastic Neighbor Embedding
PCR	Polymerase Chain Reaction
MSA	Multiple Sequence Alignment
