# Supplementary material for: Deep Learning-Enhanced Raman Microspectroscopy Enables Rapid Microbial Classification and Captures Phylogenetic Relationships
Source: Microorganisms. 2026 Jun 11;14(6):1311. doi: 10.3390/microorganisms14061311 (PMC13303631; doi:10.3390/microorganisms14061311)
Supplement: Supplementary file 1 [file microorganisms-14-01311-s001.zip › microorganisms-4295711-supplementary (1).pdf]

SUPPLEMENTARY MATERIAL

Deep Learning–Enhanced Raman Microspectroscopy Enables Rapid Microbial Classification and Captures Phylo-genetic Relationships

Beimin Liu, Zhenzhou Gu, Xianyang Xu, Weilai Lu, Tao Liu, Xueyan Gao, Xiaojing Chen, Yu Vincent Fu

This PDF file includes:

Figures S1 to S8

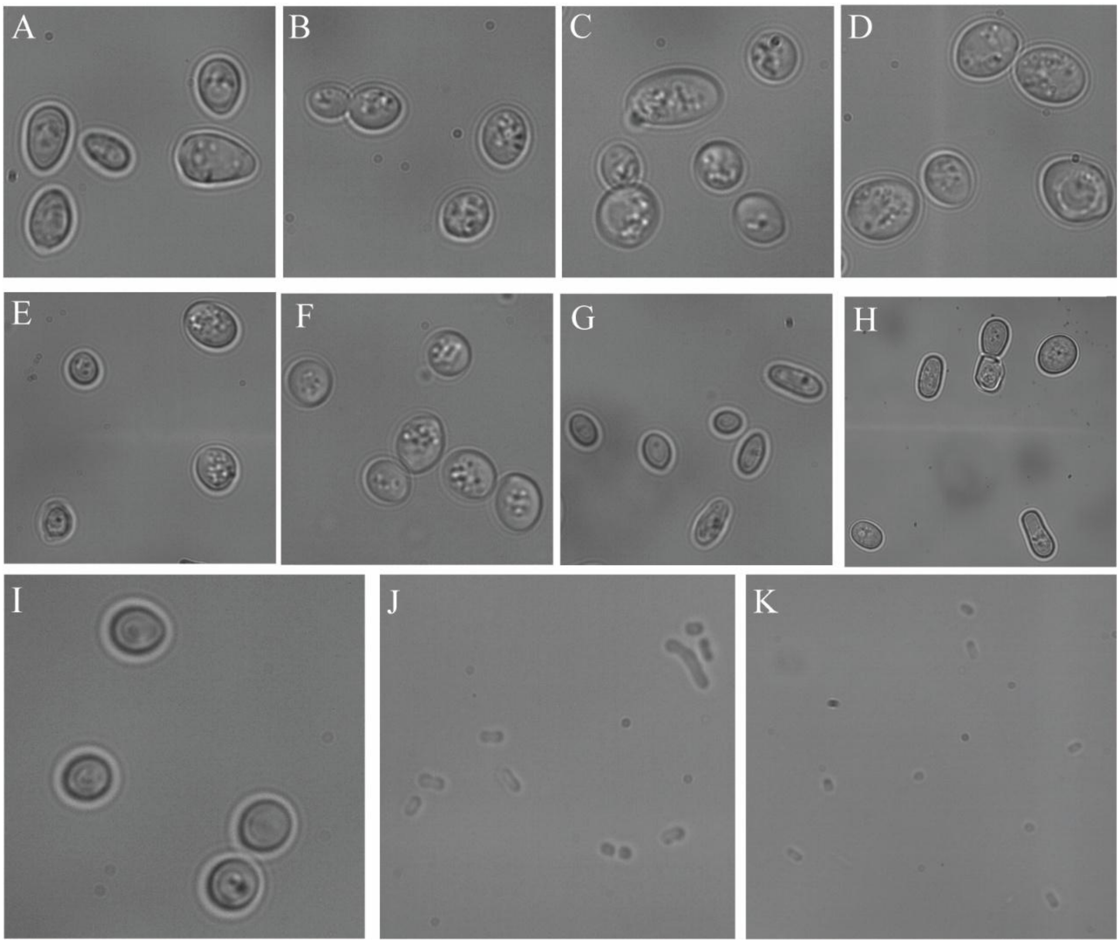

Figure S1. Morphological images of 11 different microbial strains made at 1000× magnification. (A) *S. arboricolus*; (B) *S. cerevisiae*; (C) *S. eubayanus*; (D) *S. kudriavzevii*; (E) *S. mikatae*; (F) *S. paradoxus*; (G) *P. pastoris*; (H) *G. candidum*; (I) *C. albicans*; (J) *A. baumannii*; (K) *K. pneumoniae*.

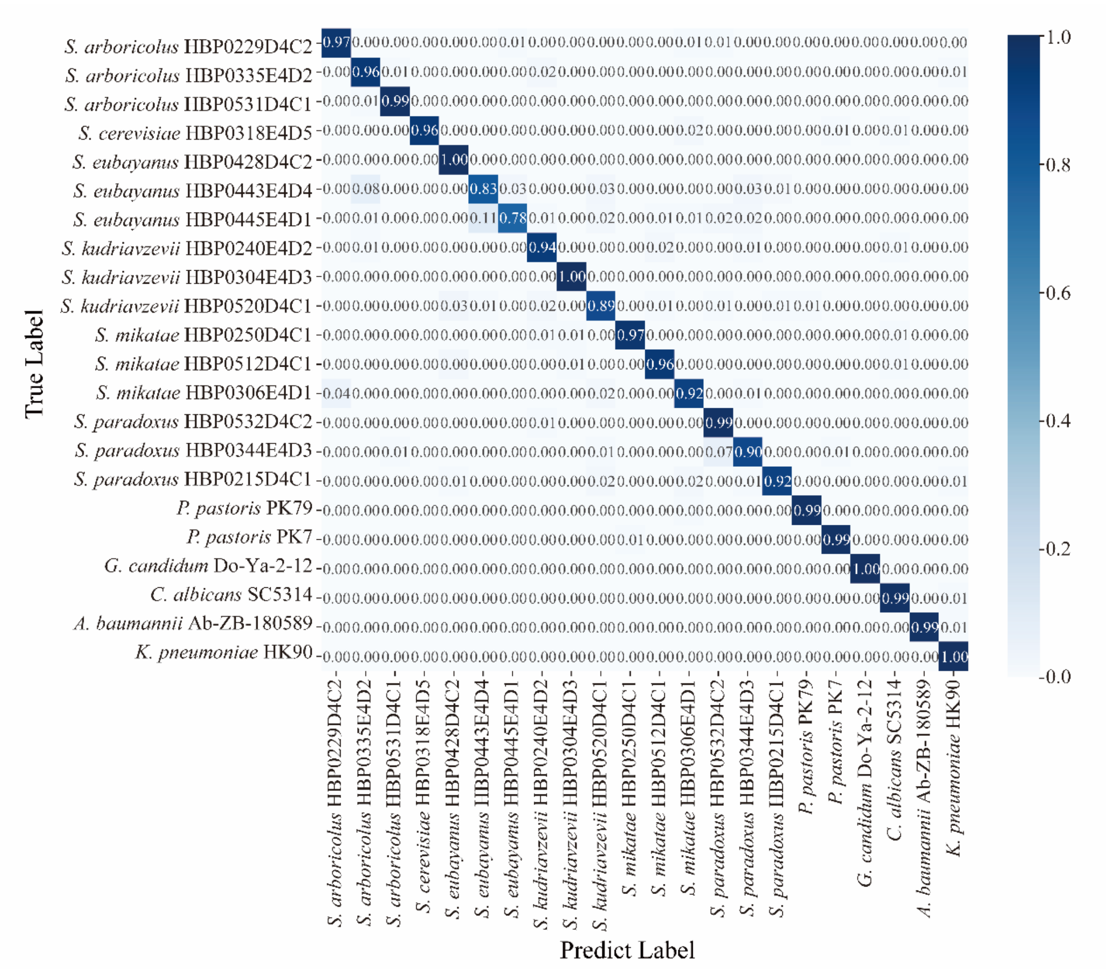

Figure S2. Test set confusion matrix of normalized Raman spectra of 22 strains based on SVM classifier, the classification accuracy reached 95.0%.

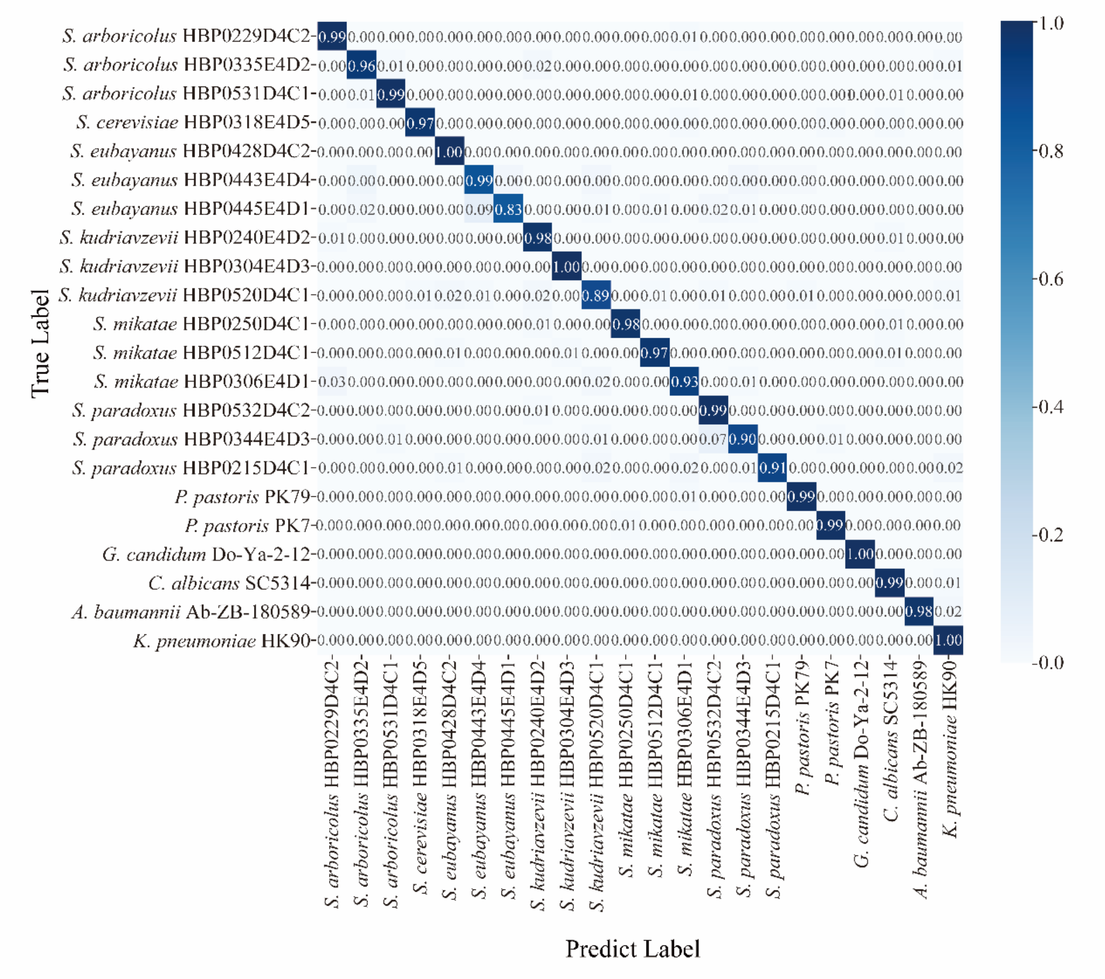

Figure S3. Test set confusion matrix of normalized Raman spectra of 22 strains based on L1 regularization preprocessing and SVM, the classification accuracy reached 95.8%.

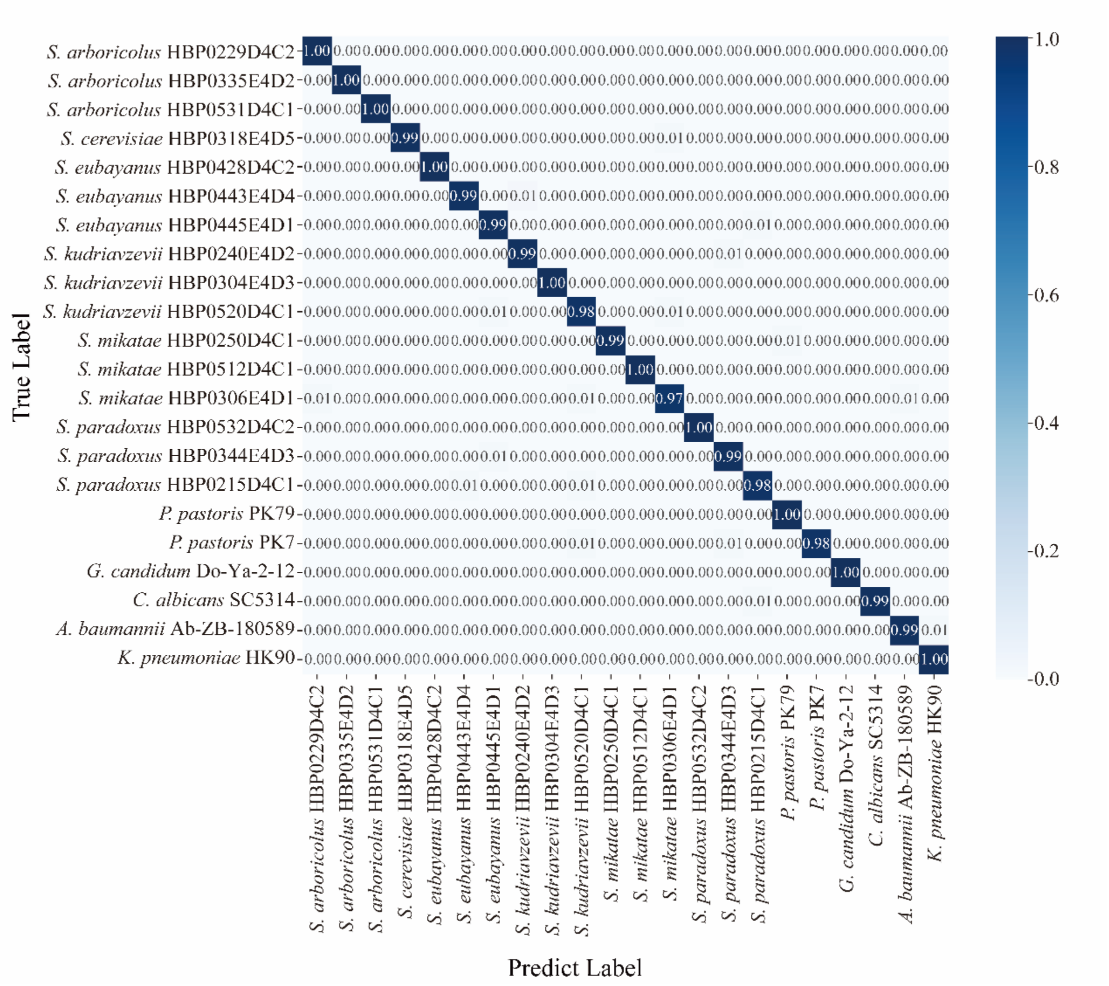

Figure S4. Test set confusion matrix of standardized Raman spectra of 22 strains based on MLP, the classification accuracy reached 99.1%.

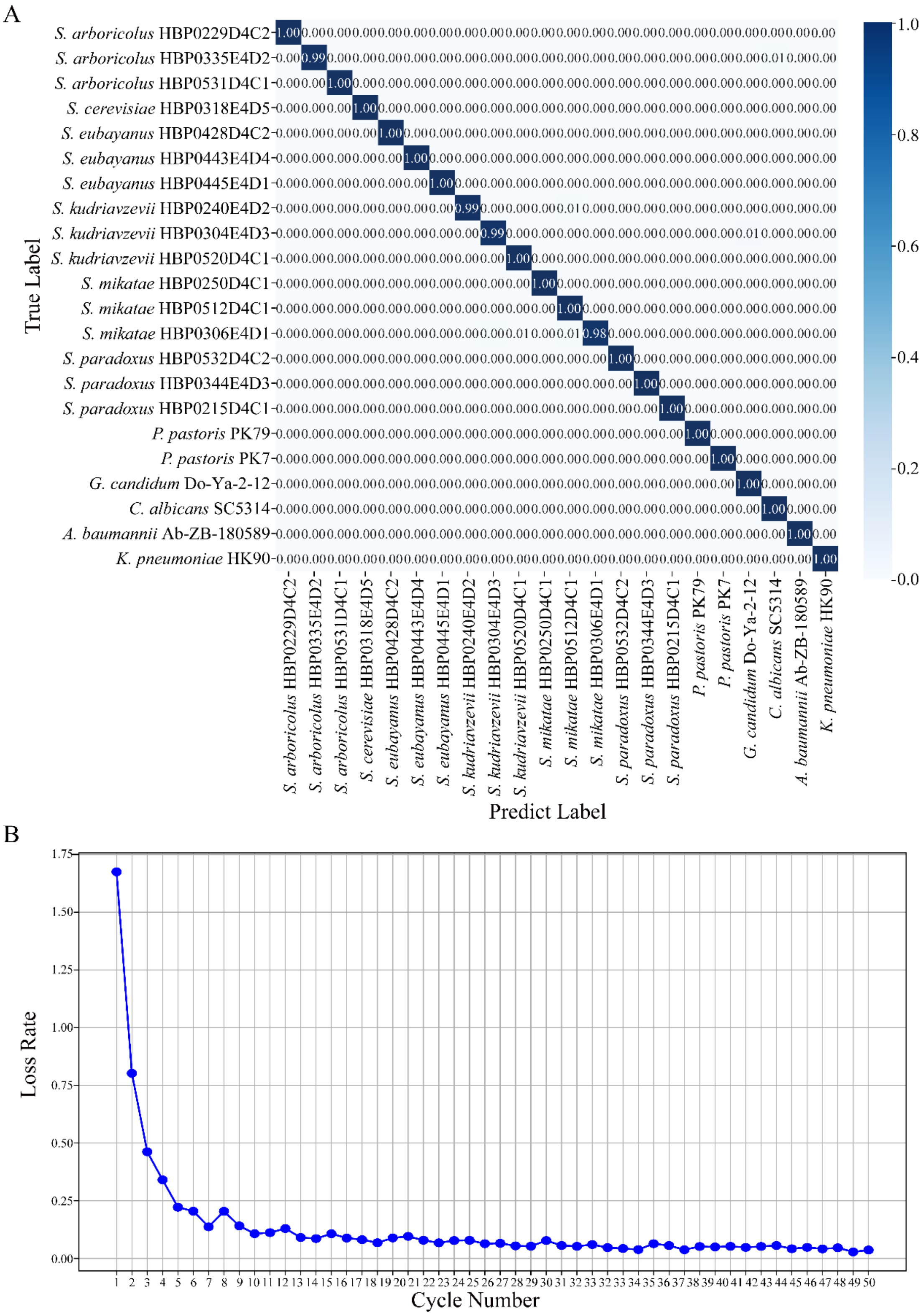

Figure S5. Test set confusion matrix and evaluation results of the 1D-CNN model. (A) Test set confusion matrix of standardized Raman spectra of 22 strains based on the 1D-CNN model, the classification accuracy reached 99.7%. (B) Loss function of the 1D-CNN model.

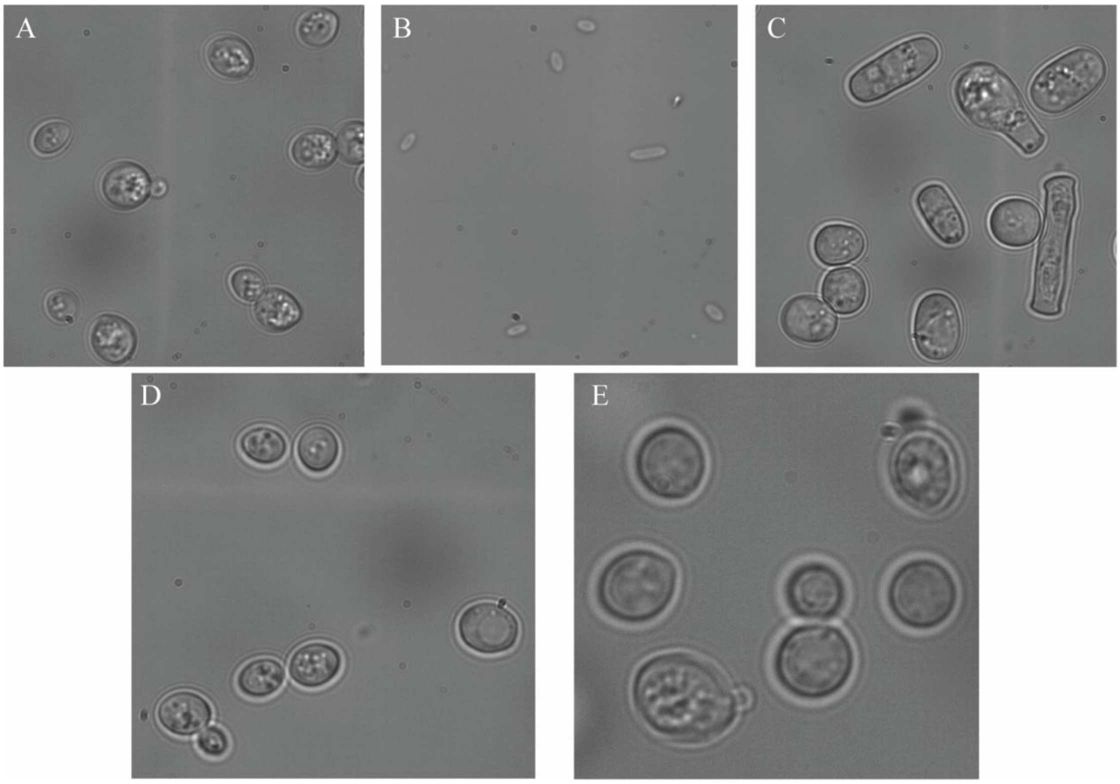

Figure S6. Morphological images of five unknown microbial strains made at 1000× magnification. (A) Sample 1; (B) Sample 2; (C) Sample 3; (D) Sample 4; (E) Sample 5.

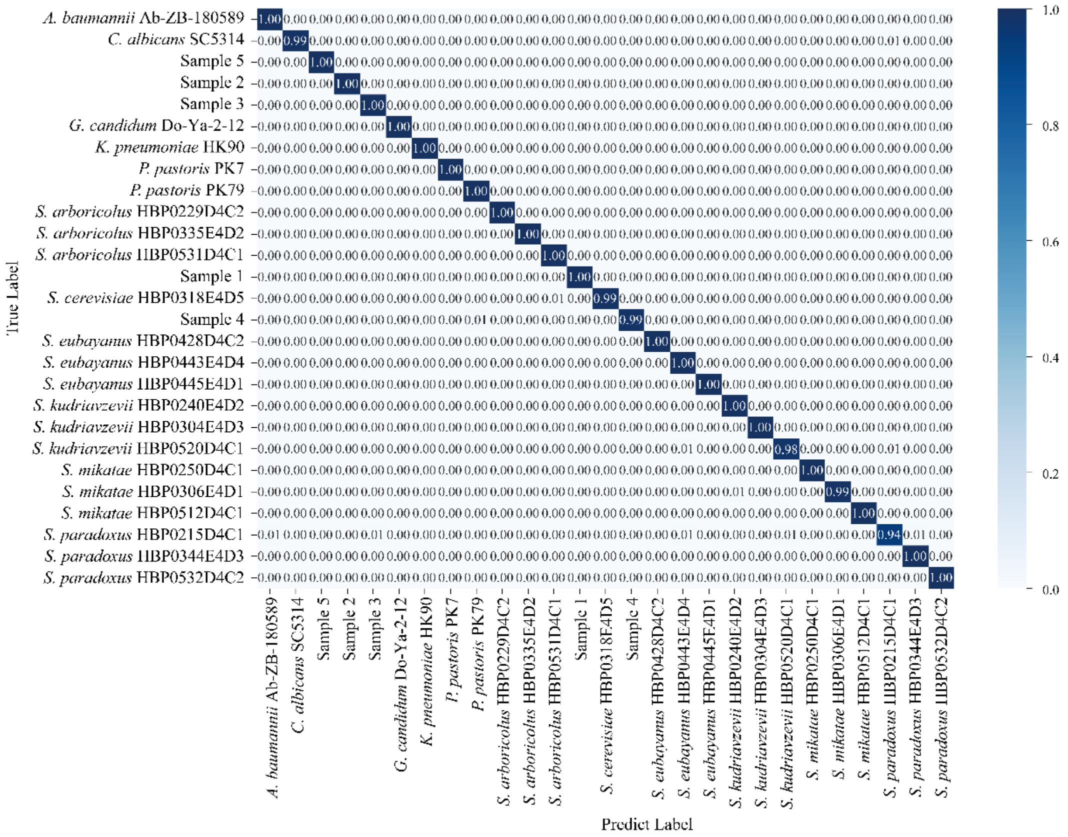

Figure S7. Classification confusion matrix results of the five unknown microbial strains on the 1D-CNN model, the classification accuracy reached 99.5%.

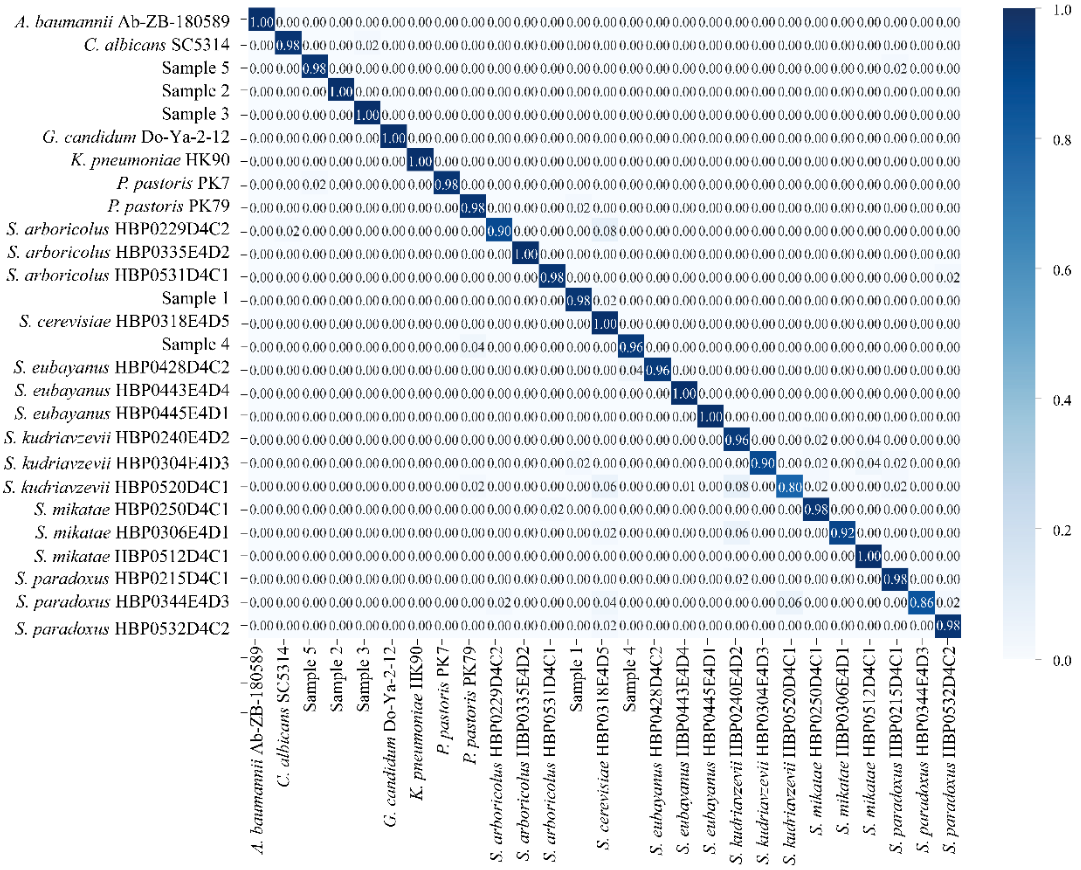

Figure S8. The classification confusion matrix results of the 27 microbial strain independent spectral test set on the 1D-CNN model show that the classification accuracy reached 94.3%.
